# Dietary Habits and Self-Reported Health Measures Among Norwegian Adults Adhering to Plant-Based Diets

**DOI:** 10.3389/fnut.2022.813482

**Published:** 2022-04-27

**Authors:** Synne Groufh-Jacobsen, Annechen Bahr Bugge, Marianne Sandsmark Morseth, Julia Tsuruta Pedersen, Sigrun Henjum

**Affiliations:** ^1^Department of Nutrition and Public Health, Faculty of Health and Sport Science, University of Agder, Kristiansand, Norway; ^2^Consumer Research Norway (SIFO), Oslo Metropolitan University, Oslo, Norway; ^3^Department of Nursing and Health Promotion, Faculty of Health Science, Oslo Metropolitan University, Oslo, Norway

**Keywords:** diet, dietary habits, flexitarian, Norway, plant-based, vegan, vegetarian, pescatarian

## Abstract

**Background:**

As plant-based diets is increasing, we aimed to investigate dietary habits, dietary motivation and self-reported health of Norwegian adults who adhere to different types of plant-based diets.

**Methods:**

In 2020, 808 subjects (530 women and 278 men) participated in an online survey, including vegans (8%), lacto-ovo vegetarians (16%), pescatarians (23%), and flexitarians (53%).

**Results:**

Half of the participants reported to consume fruits daily, three quarters consumed vegetables daily, and one quarter consumed whole grain products daily. Half of the participants reported weekly consumption of sweets and salted snacks, and 10% reported daily consumption of sugary drinks. Daily consumption of milk and dairy substitutes were reported by vegans (49%), lacto-ovo vegetarians (33%), pescatarians (32%), and flexitarians (25%). Daily consumption of meat replacement products was reported by vegans (3%), lacto-ovo vegetarians (5%), pescatarians (2%). Reported supplement use was highest among vegans (62%) and lowest among flexitarians (28%). Dietary motivations were driven by environmental (71%), health (64%), and animal welfare (49%) concerns, across all dietary groups. 75% reported their self-perceived health to be good or very good.

**Conclusion:**

We found that Norwegian adults adhering to plant-based diets consumed less fruit, vegetables, and whole grain products, together with more sugar than recommended in the Norwegian dietary guidelines. The participants reported their self-perceived health to be good or very good.

## Introduction

Use of vegetarian diets is increasing worldwide (1). However, vegan and vegetarian eating patterns are still marginal in most countries, except India (1). In Europe, the prevalence of people adhering to vegetarian diets are estimated to be between 1 and 10% (2). In Norway, the number of vegans and vegetarians was 1 and 3%, respectively, in 2018 (3). Recently, consumer demand for vegetarian food products has been increasing in Norway (3).

Plant-based diets include a variety of dietary forms. Vegans are defined as people who omit all animal source foods and may exclude honey (4). Lacto-vegetarians as those who include dairy products, but not egg products (4). Lacto-ovo vegetarians as those who include dairy and egg products (4). Pescatarians mainly have a plant-based diet, but additionally include seafood. Finally, flexitarians or semi-vegetarians can be defined as those who are trying to reduce their intake of animal source food when convenient (5), also defined as those who occasionally include meat (6).

Plant-based diets have also been associated with various health benefits (7). A previous study has shown that it is possible to secure recommended intake of nutrients in vegan and vegetarian diets (8). Meanwhile, a diet with limited amounts of animal source foods may also increase the risk of micronutrient deficiencies, such as vitamin B_12_, iodine, iron, zinc, calcium and selenium (9–13), especially if the diet is not well-planned, and enriched plant-based substitutes or supplementation is not used. Previous studies in Norway have found sufficient iron status (14) and mild-to-moderate iodine deficiency among vegans and vegetarians (12). Notably, mild-to-moderate iodine deficiency is present among the general population in Norway, but vegans are especially at risk as they omit the most important dietary iodine sources such as milk and dairy products, lean fish, and eggs (12, 15, 16).

There is global agreement that we need to act for a more sustainable food system and that consumption of plant-based foods can reduce the risk of non-communicable diseases. In addition, it has been suggested that a shift toward more plant-based diets can contribute to reduce the impact of food production on the climate and contribute to improved food security for future generations (17, 18). Thus it is important to investigate whether people who adhere to plant-based diets consume a diet aligned with the Norwegian dietary guidelines, as a diet aligned with the dietary guidelines can reduce the risk of non-communicable diseases. Although it is desirable to eat more plant-based it is also crucial that the diet is nutritionally adequate. Thus, the aim of this study was to investigate dietary habits, dietary motivation and self-reported health among Norwegian adults adhering to vegan, lacto-ovo vegetarian, pescatarian, and flexitarian diet.

## Materials and Methods

### Subjects and Participant Flow

In Norway, 81,000 respondents are registered in the Norstat panel. In the Norstat database, demographics are pre-registered to draw a representative sample of all geographical areas in Norway and other relevant characteristics, such as age, gender, and educational level. A total of 19,000 Norwegian adults were invited to participate in the survey. Subjects with omnivore-based diet or those with unfinished questionnaires were excluded. Finally, people who answered “not sure” to which diet they adhered to were excluded. As a result, 808 subjects reported adherence to a plant-based diet and thus qualified for participation. The following criteria were used in the questionnaire to define the dietary alternatives (1, 6): (1) vegan, “I omit all kinds of animal source foods”; (2) Lacto-ovo vegetarian, “I omit meat and meat products/fish and shellfish, but include milk or dairy products/eggs in my diet”; (3) pescatarian, “I omit meat and meat products, but include fish or shellfish/milk or dairy products/eggs in my diet”; (4) flexitarian, “I am trying to reduce my consumption of animal source foods when convenient”; and (5) “not sure.”

### Questionnaire

The questionnaire used in this study was developed by the Faculty of Health at OsloMet and the Norwegian Institute of Consumer Research (SIFO). Participants answered an online questionnaire, which assessed background characteristics, a food frequency questionnaire (FFQ) measuring habitual intake of selected food groups, meal habits and supplement use the previous 4 weeks. The questionnaire also assessed dietary motivation and self-reported health conditions. The FFQ was based on a previously validated questionnaire (19), which consisted of 26 selected food groups. Food consumption was reported using the following frequency options: rarely/never; monthly; weekly; and daily. Quantities were not measured. The questionnaire was pilot tested (vegan, *n* = 1 and lacto-ovo vegetarians, *n* = 5) before the study started and changed accordingly. Changes were made to adapt to vegan and vegetarian diet and relevant lifestyle factors, for example diet duration and diet motivation, including several plant-based substitutes as tofu/tempeh/seitan, plant-based substitutes for dairy products, and meat replacement products. Daily meal habits (breakfast, lunch, dinner, evening meal, and snack) were assessed with the alternatives no/yes for the previous 4 weeks. Meal habits over the past 4 weeks (restaurant visit, café visit, fast-food/take-away, canteen, and meal with friends) were assessed with the alternatives no/yes. The frequency of having homecooked meals during the previous 4 weeks was assessed with the following alternatives: never/rarely; 1–2 times per month; 1–3 times per week; 4–6 times per week; daily; and several times a day.

Supplement use was assessed using a dichotomous variable (no/yes) for use of multivitamin, omega-3 fatty acids, vitamin B_12_, vitamin D, iodine, zinc, calcium, iron, and folate. A string alternative “other supplements” was possible, if they used other types of supplements, and an alternative “do not remember.” The consumption frequency of supplements was assessed using the following alternatives: never; when convenient; rarely; and regularly.

### Assessment of Self-Reported Health

All data were based on self-reported measurements. The participants were asked to answer whatever they were suffering from for the following conditions: hypothyroidism; B_12_ deficiency; iron deficiency; vitamin D deficiency; vitamin A deficiency; zinc deficiency; calcium deficiency; reduced cholesterol; increased cholesterol; increased blood pressure. A string alternative “other conditions” was possible, as well as the alternatives “do not remember” and “none.” Self-perceived health was assessed with the following alternatives: very poor; poor; neither good or poor; good; very good; and not sure.

### Statistics

IBM SPSS version 25 (IBM Corp., Armonk, NY, United States) was used for all statistical analyses. Normality of the data was tested using the Shapiro-Wilk test, Q-Q plots, and histograms. Fisher’s exact test and Pearson’s chi-square test in crosstabulation were used to test for differences between the dietary groups using categorical variables. The Kruskal-Wallis test was used to test for differences between the dietary groups using continuous variables. The significance level used was a *p*-value < 0.05.

## Results

The participants’ background characteristics are presented in Table 1. All geographical areas in Norway were represented, 45% of participants were resident in eastern Norway. More women than men participated, 73 and 27%, respectively, no difference was found according to educational level and diet adherence. Overall, main diet motivation were environmental concerns (71%), followed by health reasons (64%), animal welfare (49%), and unnecessary killing of animals (31%), across all dietary groups.

**TABLE 1:** Characteristics of participating adult vegans, lacto-ovo vegetarians, pescatarians, and flexitarians in Norway (*n* = 808).

Characteristic	All *n* (%)	Vegans *n* (%)	Lacto-ovo vegetarians *n* (%)	Pescatarians *n* (%)	Flexitarians *n* (%)	*p*-value^a^
Participants	808 (100)	66 (8)	128 (16)	188 (23)	426 (53)	
Age^b^	40 ± 16	36 ± 13	36 ± 13	39 ± 16	43 ± 17	<0.001*
**Sex**						<0.001*
Women	530 (66)	48 (73)	106 (83)	159 (85)	217 (51)	
Man	278 (34)	18 (27)	22 (17)	29 (15)	209 (49)	
**Geographic region**						0.029*
Eastern Norway	366 (45)	19 (29)	65 (51)	96 (51)	186 (44)	
Southern Norway	87 (11)	8 (12)	13 (10)	14 (7)	52 (12)	
Mid Norway	127 (16)	18 (27)	16 (12)	29 (16)	64 (15)	
Western Norway	167 (21)	17 (26)	24 (19)	41 (22)	85 (20)	
Northern Norway	61 (7)	4 (6)	10 (8)	8 (4)	39 (9)	
Food allergy/intolerance	194 (24)	15 (23)	32 (25)	50 (27)	97 (23)	0.759
**Educational level** ^c^						0.363
<13 y education	161 (24)	16 (31)	30 (29)	34 (21)	81 (22)	
>13 y education	506 (74)	33 (65)	72 (70)	122 (77)	279 (76)	
Others	13 (2)	2 (4)	1 (1)	3 (2)	7 (2)	
**Diet adherence**						< 0.001*
<6 months	38 (5)	3 (5)	2 (2)	9 (5)	24 (6)	
6–12 months	77 (10)	5 (8)	4 (3)	9 (5)	59 (14)	
1–2 y	152 (19)	8 (12)	12 (9)	21 (11)	111 (26)	
3–4 y	142 (18)	10 (15)	28 (22)	27 (14)	77 (18)	
5–6 y	110 (14)	10 (15)	17 (13)	39 (21)	44 (10)	
>6 y	259 (32)	28 (42)	64 (50)	82 (44)	85 (20)	
Not sure	30 (4)	2 (3)	1 (1)	1 (1)	26 (6)	
**Diet motivation**						
Health reasons My appearance Dieting Environment reasons Killing of animals Animal welfare My family My friends My partner Identity reasons Taste Allergy Not sure Others^d^	519 (64) 51 (6) 81 (10) 572 (71) 248 (31) 398 (49) 60 (7) 41 (5) 59 (7) 92 (11) 76 (9) 30 (4) 9 (1) 58 (7)	44 (67) 3 (5) 3 (5) 54 (82) 48 (73) 46 (70) 7 (11) NA 8 (12) 11 (17) 12 (18) 4 (6) NA 7 (11)	76 (59) 12 (9) 9 (7) 86 (67) 75 (59) 96 (75) 12 (9) 10 (8) 6 (5) 25 (20) 23 (13) 5 (4) 1 (1) 10 (8)	112 (60) 17 (9) 9 (5) 141 (75) 73 (39) 112 (60) 16 (9) 16 (9) 13 (7) 30 (16) 25 (13) 10 (5) 3 (2) 10 (5)	287 (67) 19 (5) 60 (14) 291 (68) 52 (12) 144 (34) 25 (6) 15 (4) 32 (8) 26 (6) 16 (4) 11 (3) 5 (1) 31 (7)	0.167 0.079 0.001* 0.056 <0.001* <0.001* 0.265 0.007* 0.305 <0.001* <0.001* 0.271 0.867 0.528

^a^Test for difference: Pearson’s chi-square test, Fisher’s exact test, and Kruskal-Walli’s test; *significance < 0.05; ^b^age presented as mean ± SD; ^c^educational level missing for 128 subjects; ^d^includes animal welfare; unnecessary killing of animals; my family; environmental reasons; economic reasons; health reasons; taste; and allergy.

### Food Intake

An overview of the participants’ habitual consumption of 26 food groups is presented in Supplementary Tables 1–4.

#### Fruit and Vegetables

Half of the participants reported daily consumption of fruits and three quarters reported daily consumption of vegetables, no difference was found between the dietary groups, *p* = 0.075 and *p* = 0.077.

#### Whole Grain Foods

In total, one third reported daily consumption of whole grain products, no difference was found between the dietary groups (*p* = 0.070).

#### Nuts and Seeds

Overall, 24% reported daily consumption of nuts and seeds, 43% reported weekly consumption, and 27% reported monthly consumption. Daily consumption of nuts and seeds were highest among vegans (46%), followed by lacto-ovo vegetarians (31%), pescatarians (30%), and flexitarians (16%) (*p* = 0.001).

#### Animal Source Foods

Overall, daily consumption of meat or meat products were reported by 5%, weekly consumption by 39%, and monthly consumption by 12%. Flexitarians reported the most frequent consumption of meat or meat products (*p* ≤ 0.001). For daily consumption of milk and dairy products, the most frequent consumption were among flexitarians (56%), followed by pescatarians (47%), lacto-ovo vegetarians (36%), and vegans (2%) (*p* ≤ 0.001). Half of the participants reported weekly consumption of eggs, the highest prevalence was among flexitarians (67%), followed by pescatarians (60%) and lacto-ovo vegetarians (45%) (*p* ≤ 0.001). Further, half of the participants (49%) reported weekly consumption of fish and fish products, most commonly flexitarians (65%), followed by pescatarians (59%) and lacto-ovo vegetarians (2%) (*p* ≤ 0.001).

#### Sweets and Salted Snacks

Daily consumption of sugary drinks was reported by 10%, no difference was found between the dietary groups (*p* = 0.470). Around half of participants (52%) reported weekly consumption of sweets (including candy, ice cream, chocolate, and cake), no difference was found between the dietary groups (*p* = 0.331). Regarding salted snacks, weekly consumption was reported by half of the participants, no difference was found between the dietary groups (*p* = 0.512).

Daily consumption of artificially sweetened beverages was reported by 12%, no difference between the dietary groups (*p* = 0.162).

#### Plant-Based Food Alternatives

For daily consumption of milk and dairy substitutes, the highest prevalence was among vegans (49%), followed by lacto-ovo vegetarians (33%), pescatarians (32%), and flexitarians (25%) (*p* ≤ 0.001). For meat replacement products, 2% of the participants reported daily consumption, 25% reported weekly consumption, and 37% reported monthly consumption.

Tofu, tempeh, or seitan was consumed daily by 1% of the participants and weekly by 7%. The highest prevalence was among vegans (29%), followed by lacto-ovo vegetarians (13%), pescatarians (10%), and flexitarians (1%) (*p* ≤ 0.001).

In terms of vegetarian fast-food, 16% of the participants reported weekly consumption, with the highest prevalence among lacto-ovo vegetarians (23%), followed by pescatarians (20%), vegans (14%), and flexitarians (12%) (*p* ≤ 0.001).

#### Lentils and Legumes

Daily consumption of lentils and legumes was reported by 11% of the participants, with the highest frequency consumption among vegans (38%), followed by lacto-ovo vegetarians (21%), pescatarians (9%) and flexitarians (4%) (*p* ≤ 0.001). Regarding weekly consumption of legumes, the highest frequency consumption was among pescatarians (64%), followed by lacto-ovo vegetarians (59%), vegans (49%), and flexitarians (42%) (*p* ≤ 0.001).

#### Foods Containing Macroalgae

Less than 1% of the participants reported daily consumption of foods containing macroalgae. Weekly consumption was reported by 5%, and monthly consumption by 32%. For monthly consumption of foods containing macroalgae, the highest frequency consumption was among vegans (42%), followed by pescatarians (41%), flexitarians (29%), and lacto-ovo vegetarians (23%) (*p* ≤ 0.001).

### Meal Habits

A summary of the participant’s meal habits is presented in Table 2. The majority reported eating breakfast and lunch daily. Nearly all ate dinner daily, and about half ate evening meals daily. Furthermore, consumption of daily homecooked meals was reported by 29% of vegans, 23% of lacto-ovo vegetarians, 13% of pescatarians, and 9% of flexitarians, with a decreasing trend according to dietary strictness (*p* ≤ 0.001).

**TABLE 2:** Dietary habits and meal frequency of adult vegans, vegetarians, pescatarians, and flexitarians in Norway (*n* = 808).

	Vegans *n* = 66 *n* (%)	Lacto-ovo vegetarians *n* = 128 *n* (%)	Pescatarians *n* = 188 *n* (%)	Flexitarians *n* = 426 *n* (%)	*p*-value^a^
**Dietary habits, daily**					
Breakfast	55 (83)	99 (77)	158 (84)	350 (82)	0.477
Lunch	56 (85)	99 (77)	169 (90)	371 (87)	0.013*
Dinner	64 (97)	125 (98)	180 (96)	409 (96)	0.800
Supper	33 (50)	59 (46)	83 (44)	203 (48)	0.813
Snack	30 (46)	47 (37)	89 (47)	146 (34)	0.012*
**Dietary habits, previous week**					
Restaurant visit	21 (32)	35 (27)	53 (28)	102 (24)	0.445
Café visit	15 (23)	39 (31)	57 (30)	87 (20)	0.020*
Fast-food/Take-away	16 (24)	23 (18)	46 (25)	123 (29)	0.091
Canteen	5 (8)	13 (10)	27 (14)	64 (15)	0.243
Meal with friends	23 (35)	48 (38)	89 (47)	153 (36)	0.050*
**Homecooked dinner**					
Daily, or several times a day 4–6 times per week 1–3 times per week 1–2 times per month Never/rarely	19 (29) 31 (47) 13 (20) 2 (3) 1 (2)	30 (23) 43 (34) 34 (27) 12 (9) 9 (7)	24 (13) 86 (46) 56 (30) 14 (7) 8 (4)	40 (9) 184 (43) 135 (32) 43 (10) 24 (6)	<0.001*

^a^Test for difference: chi-square test; *significance < 0.05.

### Supplement Use

Data for supplement use is presented in Table 3. Supplement use (includes all supplements) was highest among vegans (62%), followed by pescatarians (40%), lacto-ovo vegetarians (38%), and flexitarians (28%) (*p* = 0.001). Intake/use of a multivitamin, vitamin B_12_, and vitamin D was most frequently reported by vegans. For lacto-ovo vegetarians and pescatarians, the most frequently used supplements were multivitamins, vitamin B_12_, vitamin D, and omega-3 fatty acids. Finally, among flexitarians, the most frequently reported supplements were multivitamins, omega-3 fatty acids, and vitamin D.

**TABLE 3:** Supplement use among Norwegian adults with vegan, lacto-ovo vegetarian, pescatarian, and flexitarian diets (*n* = 808).

	All *n* = 808 *n* (%)	Vegans *n* = 66 *n* (%)	Lacto-ovo vegetarians *n* = 128 *n* (%)	Pescatarians *n* = 188 *n* (%)	Flexitarians *n* = 426 *n* (%)	*p*-value^a^
**Supplement used**						
Multivitamin Omega-3 fatty acids Vitamin B_12_ Vitamin D Iodine Zinc Calcium Iron Folate Others^b^	242 (42) 290 (50) 189 (33) 260 (45) 56 (10) 35 (6) 60 (10) 125 (22) 42 (7) 98 (17)	32 (51) 21 (33) 42 (67) 33 (52) 10 (16) 4 (6) 5 (8) 12 (19) 3 (5) 5 (8)	41 (39) 44 (42) 48 (46) 57 (55) 13 (13) 6 (6) 10 (10) 32 (31) 9 (9) 13 (13)	59 (41) 75 (52) 55 (38) 57 (39) 17 (12) 12 (8) 21 (15) 41 (28) 12 (8) 26 (18)	110 (41) 150 (56) 44 (16) 113 (42) 16 (6) 13 (5) 24 (9) 40 (15) 18 (7) 54 (20)	0.476 0.004* <0.001* 0.040* 0.033* 0.573 0.293 0.001* 0.744 0.069
**Frequency of supplement use**						
Daily Often Occasionally Rarely Never	284 (35) 81 (10) 153 (19) 63 (8) 227 (28)	41 (62) 10 (15) 11 (17) 1 (2) 3 (5)	48 (38) 12 (9) 31 (24) 13 (10) 24 (19)	76 (40) 20 (11) 34 (18) 15 (8) 43 (23)	119 (28) 39 (9) 77 (18) 35 (8) 157 (37)	

^a^Crosstabulation with Pearson’s chi-square test used for difference between groups; *significance < 0.05; ^b^others include vitamin C, magnesium, vitaepro, cranberries, cod liver oil, quercetin, selenium, protein powder, medox, probiotics, nutridrink, MSM, crom, ginseng, ginger, turmeric, collagen, b-vitamins, and amino acids.

No difference was found for supplement use and residential area (*p* = 0.063) or supplement use and participant age (*p* = 0.081).

### Self-Reported Health

The participants’ self-reported health conditions, self-reported nutrient deficiency, and self-perceived health are presented in Table 4. The most frequently self-reported deficiencies were of vitamin B_12_, iron, and vitamin D. Flexitarians reported having the least self-reported deficiencies and health conditions. Overall, 75% of the participants reported that their self-perceived health was either good or very good, and only 11% reported their self-perceived health to be either poor or very poor.

**TABLE 4:** Self-reported nutrient deficiency and health conditions, and self-reported health among adults adhering to different plant-based diets in Norway (*n* = 808).

	All (*n* = 808) *n* (%)	Vegans (*n* = 66) *n* (%)	Lacto-ovo vegetarians (*n* = 128) *n* (%)	Pescatarians (*n* = 188) *n* (%)	Flexitarians (*n* = 426) *n* (%)	*p*-value*^a^*
**Nutrient deficiency and health condition (self-reported)**						
Vitamin B_12_	67 (8)	7 (11)	23 (18)	19 (10)	18 (4)	<0.001*
Thyroid disorder	16 (2)	1 (2)	4 (3)	6 (3)	5 (1)	0.208
Iron	107 (13)	16 (24)	27 (21)	40 (21)	24 (6)	<0.001*
Vitamin D	128 (16)	14 (21)	40 (31)	36 (19)	38 (9)	<0.001*
Vitamin A	4 (0.5)	NA	4 (3)	NA	NA	0.001*
Zinc	7 (1)	1 (2)	2 (2)	2 (1)	2 (1)	0.341
Calcium	7 (1)	NA	4 (3)	NA	3 (1)	0.042*
Reduced cholesterol	27 (3)	7 (11)	9 (7)	5 (3)	6 (1)	<0.001*
Increased cholesterol	8 (1)	NA	1 (1)	NA	7 (2)	0.278
Reduced blood pressure	27 (3)	5 (8)	9 (7)	5 (3)	8 (2)	0.007*
Increased blood pressure	14 (2)	NA	2 (2)	5 (3)	7 (2)	0.658
**Self-perceived health**						
Very poor	19 (2)	3 (5)	2 (2)	7 (4)	7 (2)	
Poor	69 (9)	5 (8)	10 (8)	14 (7)	40 (9)	
Neither good or poor	112 (14)	2 (3)	18 (14)	15 (8)	77 (18)	
Good	429 (53)	26 (39)	58 (45)	101 (54)	244 (57)	
Very good	177 (22)	39 (46)	38 (30)	51 (27)	58 (14)	
Not sure	2 (0.2)	NA	2 (2)	NA	NA	

^a^Test for difference: Fisher’s exact test and Pearson’s chi-square test between dietary groups using categorical variables; *significance level < 0.05.

## Discussion

In summary we found that Norwegian adults adhering to different plant-based diets consumed less fruits, vegetables, and whole grain products, and more sugary foods than recommended in the Norwegian dietary guidelines. Supplement use was highest among vegans (62%) and lowest among flexitarians (28%). Dietary motivations were driven by environmental (71%), health (64%), and animal welfare (49%) concerns, across all dietary groups. The participants reported their self-perceived health to be good or very good.

In our study, half of the participants reported not consuming fruits daily and one quarter did not consume vegetables daily, which implicate that the dietary recommendation of consuming five portions of fruits and vegetables a day is not met. However, these findings seems to be in line with the general population (20). In the Norwegian dietary guidelines, it is recommended to consume five portions of vegetables and fruits/berries daily (21). But, in our study, we were not able to evaluate if the participants who reported daily intake of fruits and vegetables secured the recommended intake, as the frequency option “daily” spanned 1–7 times a day. Therefore, the daily consumption of fruits and vegetables recorded in our study does not necessarily imply that the participants met the recommended intake. A systematic review (22), found higher dietary quality among vegetarians compared to non-vegetarians, as vegetarians reported higher fruit and vegetable intake. Another study (23) also reported higher fruit and vegetable intake among adolescent vegetarians compared to non-vegetarians. In our study, there was no difference between the dietary groups for fruit and vegetable consumption.

In the Norwegian dietary guidelines, it is recommended to consume whole grain foods every day (21), while in our study, only one quarter of the participants consumed whole grain foods daily, which indicate that the recommendation of daily consumption of whole grain foods are not met for the majority of the participants. Further research is needed to investigate fruit, vegetable and whole grain intake among people adhering to different plant-based diets compared to non-vegetarians.

In our study, consumption of dairy substitutes was reported by half of the vegans and by one quarter of the lacto-ovo vegetarians, pescatarians and flexitarians. For meat substitutes, daily consumption was low (2%), across all diet groups. A newly published study found that the nutritionally component of dairy and meat substitutes on the Norwegian market vary (24). In France, a study found (25) that vegans and vegetarians had poorer diet quality due to higher intake of ultra-processed foods, which plant-based replacements products are often classified as. Another study by Belardo et al. (26), highlights in a clinical practice statement that the beneficial effects of vegetarian diets depends on what foods that are consumed, whether the diet consist of fruits, vegetables, nuts, legumes, whole grains, and vegetable oils, or if the diet mainly consist of high energy density foods, it can have either health promoting or unhealthy health effects. The intake of ultra-processed foods was not assessed in our study; however, few participants reported consuming homecooked meals daily, which suggests a higher consumption of pre-prepared foods (27, 28). Further studies are needed to evaluate the diet quality of people adhering to plant-based diets compared to people with a non-vegetarian diet.

Previous studies have proposed two main motivations for people who want to reduce their intake of meat, those who are motivated by health and those by animal welfare (29). Environmental concerns have been pointed out as one of the main motivational factors, however this has been proposed to account for a smaller fraction of vegetarians (29). In our study, environmental concerns were found to be the main motivation for participants to consume less meat. Secondly, health and then animal welfare were reported as other main diet motivations. This is consistent with previous findings in Norway (30), where 60% reported having altered their dietary habits due to environmental concerns over the past years. A previous study (31) found that vegans and vegetarians were more motivated by environmental concerns than semi-vegetarians, in terms of adhering to a plant-based diet. This finding was not reflected in our study, as we did not find any difference between the diet groups. A possible reason for this finding is that there has been a lot of media attention over the past years on climate and environmental changes. Diet motivation might influence eating behavior, however, this was not investigated in our study. As previous studies have suggested that the eating behaviors of vegetarians who are ethically motivated, health motivated or environmentally motivated differs (32, 33), further studies should investigate how diet motivation influence eating behavior of people adhering to different plant-based diets.

In our study, few reported having experienced health related conditions or nutrient deficiencies. The most frequently self-reported deficiencies were vitamin B_12_, iron, and vitamin D. Previous studies have reported a higher risk of deficiencies in people with restrictive diets, especially iron, iodine, and vitamin B_12_ (9–11). A previous study in Norway revealed mild-to-moderate iodine deficiency among vegans and vegetarians, but sufficient iron status (12, 14). However, studies in Norway have also shown that several population groups in the general population are at risk of iodine deficiency (15, 16). Norway is one of the few countries that still has not initiated mandatory iodine fortification; therefore, dietary iodine sources as dairy products, lean fish and eggs are crucial to ensure adequate iodine status. The lack of access to adequately fortified salt in Norway is especially challenging for vegans who thus rely on consumption of iodine supplements or macroalgae. In our study, a low percentage (10%) of participants reported iodine supplement use, which may suggest inadequate iodine intake among our participants, especially among vegans who omit the most important dietary iodine sources as milk and dairy products, lean fish and eggs. Nearly half of the participants in our study reported daily supplement use (includes all supplements), with the highest daily supplement use among vegans and the lowest among flexitarians. It is important that people adhering to a vegetarian diet, especially a vegan diet, are educated about the appropriate supplements needed to avoid nutrient deficiency. In our study, overall B12 supplement use was low (33%), however, a high percentage of vegans (67%) and vegetarians (46%) reported B12 supplement use. For omega-3 fatty acids supplement use, less than half of the vegans and vegetarians reported supplement use and around half reported vitamin-D supplement use. Below one quarter reported use of iron and calcium supplements.

In our study, high proportion of the participants were women, higher educated, and living in urban areas, consistent with previous studies on people consuming plant-based diets (29, 34, 35). The educational level in our study was almost twice as high as in the general population in Norway (36). In addition, a high proportion of our respondents were resident in the Oslo area (the capital of Norway), which is consistent with previous findings (12, 30). Another limitation is the categorization of participants into the dietary groups, which was based on self-reported dietary practice. In addition, we defined “flexitarians” as those trying to reduce their intake of animal source food. As previously mentioned a clearer definition of flexitarians is needed (6). Regarding use of self-reported dietary data and self-reported health, our data would have been strengthened by using blood biomarkers and urine samples to evaluate micronutrient status. There is also a risk of under -or over reported intake of foods when using self-reported data. This study was also conducted during the COVID-19 pandemic, which might have had an impact on what they ate, how they prepared the food and where they ate the food. In a newly study in Norway (37), they found that distress in relation to the pandemic could impact the consumption of high sugary foods and beverage, our study would have been strengthened by including items concerning if their diet had been altered during the COVID-19 pandemic.

## Conclusion

We found that Norwegian adults adhering to different plant-based diets consumed less fruits, vegetables, and whole grain products, and more sugar than recommended in the Norwegian dietary guidelines. The participants reported their self-perceived health to be good or very good.

Further studies should be conducted to evaluate the diet quality of people adhering to different types of plant-based diets, if they are able to replace animal source foods with nutritionally adequate foods and if the diet is aligned with the dietary guidelines, compared to a control group of non-vegetarians.

## Data Availability Statement

The original contributions presented in the study are included in the article/Supplementary Material, further inquiries can be directed to the corresponding author/s.

## Ethics Statement

The study involved human participants and were reviewed and approved by the Norwegian Center for Research Data/NSD/708529. The participants provided their written informed consent to participate in this study.

## Author Contributions

SH and AB: conceptualization and project administration. SG-J: formal analysis and writing—original draft preparation. SG-J, SH, and AB: investigation. SG-J, SH, AB, MM, and JP: writing—review and editing. All authors have read and agreed to the published version of the manuscript.

## Conflict of Interest

The authors declare that the research was conducted in the absence of any commercial or financial relationships that could be construed as a potential conflict of interest.

## Publisher’s Note

All claims expressed in this article are solely those of the authors and do not necessarily represent those of their affiliated organizations, or those of the publisher, the editors and the reviewers. Any product that may be evaluated in this article, or claim that may be made by its manufacturer, is not guaranteed or endorsed by the publisher.
