# Supplementary material for: Dietary Habits and Self-Reported Health Measures Among Norwegian Adults Adhering to Plant-Based Diets
Source: Front Nutr. 2022 Apr 27;9:813482. doi: 10.3389/fnut.2022.813482 (PMC9094011; doi:10.3389/fnut.2022.813482)
Supplement: Supplementary file 1 [file Data_Sheet_1.docx]

Supplementary Material

# Supplementary Tables

**Supplemental Table 1** Consumption frequency of 26 selected food groups among adult vegans (n=66)

| Food group | Never/rarely  *n* (%) | Monthly  *n* (%) | Weekly  *n* (%) | Daily  *n* (%) |
| --- | --- | --- | --- | --- |
| Grain products (White) | 10 (15) | 21 (32) | 25 (38) | 10 (15) |
| Grain products (Full grain) | 1 (2) | 10 (15) | 25 (38) | 30 (46) |
| Couscous, bulgur, and quinoa | 13 (20) | 28 (42) | 25 (38) | 0 |
| Lentils, beans, and chickpeas | 2 (3) | 7 (11) | 32 (49) | 25 (38) |
| Vegetables, all types | 1 (2) | 1 (2) | 7 (11) | 57 (86) |
| Fruits and berries, all types | 1 (2) | 5 (8) | 17 (26) | 43 (65) |
| Potatoes, all types | 3 (5) | 21 (32) | 40 (61) | 2 (3) |
| Nuts and seeds, all types | 1 (2) | 9 (14) | 26 (39) | 30 (46) |
| Rice and rice products | 5 (8) | 23 (35) | 35 (53) | 3 (5) |
| Tofu, tempeh, and seitan | 9 (14) | 36 (55) | 19 (29) | 2 (3) |
| Milk/cream/cheese replacement | 7 (11) | 3 (4) | 24 (36) | 32 (49) |
| Meat replacement | 7 (11) | 28 (42) | 29 (44) | 2 (3) |
| Meat or meat products | 65 (99) | 1 (1) | 0 | 0 |
| Macroalgae | 33 (50) | 28 (42) | 4 (6) | 1 (2) |
| Fish and seafood | 59 (89) | 7 (11) | 0 | 0 |
| Vegetarian fast-food | 24 (36) | 32 (49) | 9 (14) | 1 (2) |
| Milk and dairy products | 58 (88) | 6 (9) | 1 (2) | 1 (2) |
| Eggs | 63 (96) | 2 (3) | 1 (2) | 0 |
| Smoothie | 18 (27) | 23 (35) | 18 (27) | 7 (11) |
| Sweets (cake, chocolate, candy) | 11 (17) | 25 (38) | 29 (44) | 1 (2) |
| Salted snacks | 10 (15) | 19 (29) | 37 (56) | 0 |
| Oil, cooking | 4 (6) | 7 (11) | 24 (36) | 31 (47) |
| Sweetened beverage | 24 (36) | 21 (32) | 18 (27) | 3 (5) |
| Artificially sweetened beverage | 38 (58) | 11 (15) | 14 (21) | 4 (6) |
| Coffee and tea | 6 (9) | 5 (8) | 6 (9) | 49 (74) |
| Alcohol | 26 (39) | 21 (32) | 18 (27) | 1 (2) |

**Supplemental Table 2** Consumption frequency of 26 selected food groups among adult lacto-ovo vegetarians (n=128)

| Food group | Never/rarely  *n* (%) | Monthly  *n* (%) | Weekly  *n* (%) | Daily  *n* (%) |
| --- | --- | --- | --- | --- |
| Grain products (White) | 12 (9) | 26 (20) | 63 (49) | 27 (21) |
| Grain products (Full grain) | 5 (4) | 18 (14) | 51 (40) | 54 (42) |
| Couscous, bulgur, and quinoa | 36 (28) | 56 (44) | 33 (26) | 3 (2) |
| Lentils, beans, and chickpeas | 6 (5) | 19 (15) | 76 (59) | 27 (21) |
| Vegetables, all types | 0 | 4 (3) | 24 (19) | 100 (78) |
| Fruits and berries, all types | 4 (3) | 14 (11) | 51 (40) | 59 (46) |
| Potatoes, all types | 19 (15) | 50 (39) | 57 (45) | 2 (2) |
| Nuts and seeds, all types | 9 (7) | 32 (25) | 48 (38) | 39 (31) |
| Rice and rice products | 6 (5) | 59 (46) | 60 (47) | 3(2) |
| Tofu, tempeh, and seitan | 59 (46) | 52 (41) | 16 (13) | 1 (1) |
| Milk/cream/cheese replacement | 19 (15) | 26 (20) | 41 (32) | 42 (33) |
| Meat replacement | 16 (13) | 53 (41) | 53 (41) | 6 (5) |
| Meat or meat products | 121 (95) | 5 (3) | 2 (2) | 1 (1) |
| Macroalgae | 93 (73) | 30 (23) | 4 (3) | 1 (1) |
| Fish and seafood | 116 (91) | 9 (7) | 2 (2) | 0 |
| Vegetarian fast-food | 32 (25) | 64 (50) | 30 (23) | 2 (2) |
| Milk and dairy products | 6 (5) | 26 (20) | 50 (39) | 46 (36) |
| Eggs | 23 (18) | 35 (27) | 57 (45) | 13 (10) |
| Smoothie | 56 (44) | 48 (38) | 19 (15) | 5 (4) |
| Sweets (cake, chocolate, candy) | 15 (12) | 48 (38) | 59 (46) | 6 (5) |
| Salted snacks | 17 (13) | 48 (38) | 59 (46) | 4 (3) |
| Oil, cooking | 9 (7) | 9 (7) | 62 (48) | 48 (38) |
| Sweetened beverage | 46 (36) | 36 (28) | 31 (24) | 14 (12) |
| Artificially sweetened beverage | 60 (47) | 18 (14) | 27 (21) | 23 (18) |
| Coffee and tea | 13 (10) | 7 (6) | 24 (19) | 84 (66) |
| Alcohol | 52 (41) | 45 (35) | 24 (19) | 7 (6) |

**Supplemental Table 3** Consumption frequency of 26 selected food groups among adult pescatarians (n=188)

| Food group | Never/rarely  *n* (%) | Monthly  *n* (%) | Weekly  *n* (%) | Daily  *n* (%) |
| --- | --- | --- | --- | --- |
| Grain products (White) | 14 (7) | 46 (25) | 92 (49) | 36 (19) |
| Grain products (Full grain) | 5 (3) | 19 (10) | 61 (32) | 103 (55) |
| Couscous, bulgur, and quinoa | 56 (30) | 95 (51) | 36 (19) | 1 (1) |
| Lentils, beans, and chickpeas | 10 (5) | 40 (21) | 121 (64) | 17 (9) |
| Vegetables, all types | 1 (1) | 4 (2) | 35 (19) | 148 (79) |
| Fruits and berries, all types | 1 (1) | 20 (11) | 62 (33) | 105 (56) |
| Potatoes, all types | 16 (9) | 71 (38) | 94 (50) | 7 (4) |
| Nuts and seeds, all types | 15 (8) | 41 (22) | 76 (40) | 56 (30) |
| Rice and rice products | 14 (7) | 79 (42) | 94 (50) | 1 (1) |
| Tofu, tempeh, and seitan | 111 (59) | 59 (31) | 18 (10) | 0 |
| Milk/cream/cheese replacement | 52 (28) | 33 (18) | 43 (23) | 60 (32) |
| Meat replacement | 36 (19) | 79 (42) | 69 (37) | 4 (2) |
| Meat or meat products | 170 (90) | 12 (6) | 6 (3) | 0 |
| Macroalgae | 96 (51) | 77 (41) | 14 (7) | 1 (1) |
| Fish and seafood | 16 (9) | 51(27) | 111 (59) | 10 (5) |
| Vegetarian fast-food | 62 (33) | 87 (46) | 38 (20) | 1 (1) |
| Milk and dairy products | 15 (8) | 18 (10) | 66 (35) | 89 (47) |
| Eggs | 12 (6) | 37 (20) | 112 (60) | 27 (14) |
| Smoothie | 70 (37) | 74 (39) | 36 (19) | 8 (4) |
| Sweets (cake, chocolate, candy) | 15 (8) | 64 (34) | 100 (53) | 9 (5) |
| Salted snacks | 34 (18) | 64 (34) | 85 (45) | 5 (3) |
| Oil, cooking | 9 (2) | 44 (10) | 219 (51) | 154 (36) |
| Sweetened beverage | 64 (34) | 61 (32) | 49 (26) | 14 (7) |
| Artificially sweetened beverage | 42 (78) | 43 (23) | 48 (26) | 19 (10) |
| Coffee and tea | 17 (9) | 7 (4) | 24 (13) | 140 (75) |
| Alcohol | 65 (35) | 51 (27) | 70 (37) | 2 (1) |

**Supplemental Table 4** Consumption frequency of 26 selected food groups among adult flexitarians (n=426)

| Food group | Never/rarely  *n* (%) | Monthly  *n* (%) | Weekly  *n* (%) | Daily  *n* (%) |
| --- | --- | --- | --- | --- |
| Grain products (White) | 29 (7) | 97 (23) | 212 (50) | 88 (21) |
| Grain products (Full grain) | 17 (4) | 50 (12) | 184 (43) | 175 (41) |
| Couscous, bulgur, and quinoa | 200 (47) | 181 (43) | 42 (10) | 3 (1) |
| Lentils, beans, and chickpeas | 54 (13) | 177 (42) | 177 (42) | 18 (4) |
| Vegetables, all types | 2 (1) | 12 (3) | 114 (27) | 298 (70) |
| Fruits and berries, all types | 6 (1) | 41 (10) | 180 (42) | 199 (47) |
| Potatoes, all types | 30 (7) | 113 (27) | 250 (59) | 33 (8) |
| Nuts and seeds, all types | 32 (8) | 132 (31) | 193 (45) | 69 (16) |
| Rice and rice products | 33 (8) | 154 (36) | 224 (53) | 15 (4) |
| Tofu, tempeh, and seitan | 361 (85) | 56 (13) | 6 (1) | 3 (1) |
| Milk/cream/cheese replacement | 171 (40) | 47 (11) | 101 (24) | 107 (25) |
| Meat replacement | 236 (55) | 137 (32) | 53 (12) | 0 |
| Meat or meat products | 6 (1) | 78 (19) | 307 (72) | 35 (8) |
| Macroalgae | 283 (66) | 125 (29) | 16 (4) | 2 (1) |
| Fish and seafood | 37 (9) | 96 (23) | 278 (65) | 15 (4) |
| Vegetarian fast-food | 225 (53) | 147 (35) | 51 (12) | 3 (1) |
| Milk and dairy products | 15 (4) | 39 (9) | 132 (31) | 240 (56) |
| Eggs | 20 (5) | 60 (14) | 287 (67) | 59 (14) |
| Smoothie | 197 (46) | 147 (35) | 68 (16) | 14 (3) |
| Sweets (cake, chocolate, candy) | 33 (8) | 147 (35) | 230 (54) | 16 (4) |
| Salted snacks | 51 (12) | 158 (37) | 205 (48) | 12 (3) |
| Oil, cooking | 9 (2) | 44 (10) | 219 (51) | 154 (36) |
| Sweetened beverage | 136 (32) | 124 (29) | 120 (28) | 46 (11) |
| Artificially sweetened beverage | 185 (43) | 75 (18) | 119 (28) | 47 (11) |
| Coffee and tea | 34 (8) | 25 (6) | 50 (12) | 317 (74) |
| Alcohol | 106 (25) | 131 (31) | 175 (41) | 14 (3) |
